# Impact of Timing of Influenza Vaccination in Pregnancy on Transplacental Antibody Transfer, Influenza Incidence, and Birth Outcomes: A Randomized Trial in Rural Nepal

**DOI:** 10.1093/cid/ciy090

**Published:** 2018-02-14

**Authors:** Joanne Katz, Janet A Englund, Mark C Steinhoff, Subarna K Khatry, Laxman Shrestha, Jane Kuypers, Luke C Mullany, Helen Y Chu, Steven C LeClerq, Naoko Kozuki, James M Tielsch

**Affiliations:** 1 Johns Hopkins Bloomberg School of Public Health, Department of International Health, Baltimore, Maryland; 2 Seattle Children’s Hospital and Research Foundation, University of Washington, Seattle; 3 Global Health Center, Cincinnati Children’s Hospital Medical Center, Ohio; 4 Nepal Nutrition Intervention Project – Sarlahi, Nepal; 5 Tribhuvan University, Department of Pediatrics and Child Health, Institute of Medicine, Kathmandu, Nepal; 6 School of Medicine, University of Washington, Molecular Virology Laboratory, Seattle; 7 University of Washington, Harborview Medical Center, Seattle; 8 Milken Institute School of Public Health, George Washington University, Department of Global Health, Washington, D.C

**Keywords:** influenza vaccine, pregnancy, birth weight, transplacental antibody, influenza incidence

## Abstract

**Background:**

Maternal influenza vaccination protects mothers and their infants in low resource settings, but little is known about whether the protection varies by gestational age at vaccination.

**Methods:**

Women of childbearing age in rural southern Nepal were surveilled for pregnancy, consented and randomized to receive maternal influenza vaccination or placebo, with randomization stratified on gestational age (17–25 or 26–34 weeks). Enrollment occurred in 2 annual cohorts, and vaccinations occurred from April 2011 through September 2013.

**Results:**

In sum, 3693 women consented and enrolled, resulting in 3646 live births. Although cord blood antibody titers and the rise in maternal titers were generally greater when women were vaccinated later in pregnancy, this was not statistically significant. The incidence risk ratio (IRR) for maternal influenza in pregnancy through 6 months postpartum was 0.62 (95% confidence interval [CI]: 0.35, 1.10) for those vaccinated 17–25 weeks gestation and 0.89 (95% CI: 0.39, 2.00) for those 26–34 weeks. Infant influenza IRRs were 0.73 (95% CI: 0.51, 1.05) for those whose mothers were vaccinated earlier in gestation, and 0.63 (95% CI: 0.37, 1.08) for those later. Relative risks (RR) for low birthweight were 0.83 (95% CI: 0.71, 0.98) and 0.90 (95% CI: 0.72, 1.12) for 17–25 and 26–34 weeks gestation at vaccination, respectively. IRRs did not differ for small-for-gestational age or preterm. No RRs were statistically different by timing of vaccine receipt.

**Conclusions:**

Vaccine efficacy did not vary by gestational age at vaccination, making maternal influenza immunization programs easier to implement where women present for care late in pregnancy.

**Clinical Trials Registration:**

NCT01034254

## Teaser

This placebo-controlled randomized trial in rural Nepal found that maternal influenza vaccination in either the second or third trimester of pregnancy provided similar birth outcomes and protection from influenza in infancy.

Influenza in pregnancy, particularly pandemic influenza, has been shown to cause more severe illness and hospitalization in pregnant women than in the nonpregnant population [1, 2]. Influenza vaccination is recommended by the World Health Organization (WHO) for pregnant women, although vaccination is not standard policy in many low and middle income countries [3]. Reasons for this vary but include logistical and financial constraints, as well as a lack of evidence for influenza burden in some countries. Recent randomized trials of influenza vaccine in pregnancy in low income settings have demonstrated a protective effect against maternal and infant influenza [4–7] as well as increased birthweight in South Asia [7, 8]. Maternal vaccination in pregnancy provides the potential for protecting the infant early in life, but evidence on timing of influenza vaccination in pregnancy on efficacy in infants is lacking [9]. Maternal influenza vaccination earlier in pregnancy may provide protection in infants born preterm and potentially more time for protection of the mother against illness during pregnancy, thereby perhaps improving birth outcomes. However, vaccination in the third trimester may lead to higher levels of antibody transfer from the mother to the infant and hence improve protection against illness early in life. One study examining maternal and cord blood antibody response to the H1N1 vaccine at delivery found a lower level of antibodies for those vaccinated earlier in pregnancy [10] but another study found comparable antibody levels [11]. Neither examined protection from influenza in the infants. The South African maternal influenza vaccine trial found higher levels of transplacental transfer associated with a longer interval between vaccination and delivery in HIV uninfected women [12]. In low resource settings, the number of antenatal care (ANC) visits is increasing, but early ANC attendance is still low. Therefore it would be useful for governments considering implementing a maternal influenza vaccination program to know whether the timing of vaccine delivery in pregnancy is important to increase efficacy in infants.

We conducted an individually randomized population-based trial of maternal influenza vaccination in rural southern Nepal from April 2011 through September 2013 and estimated the efficacy of the vaccine among infants from birth through 6 months of age [7]. We used the data from this trial to examine whether vaccine efficacy varied by gestational age at vaccination.

## METHODS

Details of the trial methods are presented elsewhere [7, 13, 14]. Briefly, a census of women of childbearing age (15–40 years) was conducted in 9 Village Development Committees (VDC) of rural Sarlahi district in the low lying plains of Nepal, bordering Bihar, India. Women who identified themselves as pregnant and of gestational age between 17 and 34 weeks were offered enrollment in the trial. Women not pregnant at that time were visited every 5 weeks and asked if they had had a period since the last visit. If yes, the date of last menstrual period (LMP) was recorded. If not, they were offered a pregnancy test and if positive were consented into the trial. Hence prevalent and incident pregnancies were enrolled in the trial. Women were enrolled in 2 distinct annual cohorts. Cohort 1 was enrolled from April 25, 2011, through April 24, 2012, and cohort 2 from April 25, 2012, through April 24, 2013. Pregnant women between 17 and 34 weeks gestation were individually randomized to receive the currently recommended trivalent inactivated influenza vaccine as early as possible in pregnancy within this gestational window. Randomization was conducted in blocks of 8, stratified by VDC and gestational age (17–25 weeks and 26–34 weeks) within VDC, based on date of LMP obtained from the 5 weekly surveillance. This resulted in balanced numbers of women in each stratum of gestational age, but with most women enrolled and vaccinated early in pregnancy (except for the prevalent pregnancies). To better address the question of whether timing of maternal vaccination altered efficacy, the number of women vaccinated later in pregnancy was increased in the second cohort (which were all incident pregnancies identified prior to 17 weeks gestation) by randomizing them to vaccine or placebo as in cohort 1 but randomly assigning the timing of vaccine receipt to be spread evenly over 17–34 weeks gestation. The assignment to vaccine or saline placebo continued to follow the randomization strategy used in the first cohort (blocked by 8, stratified by VDC and 17–25 vs 26–34 weeks gestation).

The primary outcomes of the original trial were maternal influenza-like illness (ILI), laboratory confirmed influenza in infants, and low birth weight (LBW). There were multiple secondary outcomes including preterm birth, maternal laboratory confirmed influenza and others [7, 14]. To address whether vaccine timing in pregnancy alters efficacy, this secondary analysis examined the following outcomes: the rise in geometric mean of the hemagglutination-inhibition antibody (HAI) titers to influenza antigens obtained from serum samples taken prior to vaccination and within the first week postpartum, mean influenza HAI titers in cord blood, maternal to infant antibody transfer ratio, maternal seroconversion and seroprotection, laboratory confirmed influenza in women during pregnancy and through 180 days postpartum, laboratory confirmed influenza in infants from birth through 180 days of age, and prevalence of low birth weight (<2500 g taken as soon after birth as possible but no later than 72 hours after birth).

Two different vaccine formulations were used in the trial. These were the recommended vaccines for seasonal use in northern and southern hemispheres during those time periods. Between April 25, 2011, and October 15, 2012, a vaccine containing A/H3N2 Perth, A/H1N1 California, and B Brisbane (Victoria) was used, and from October 2012 to September 2013 the vaccine contained A/H3N2 Victoria, A/H1N1 California, and B Wisconsin (Yamagata). The first vaccine formulation covered all of the 1st annual cohort and part of the 2nd annual cohort trial periods.

The study proposed to collect blood samples from a subset of participants to confirm seroprotection and seroconversion. Ultimately, a convenience blood sample of 208 women-infant pairs was collected prior to vaccination, at 1 week and 3 months postpartum, and cord blood was collected from the infant after delivery. HAI assays were used to test for antibodies to influenza antigens contained in the vaccines by a CLIA certified laboratory at Cincinnati Children’s Hospital. Laboratory staff were blinded to the vaccine assignments and timing of vaccine. Maternal samples were collected prior to vaccination, at home visits postpartum, and cord blood was collected at facilities or home (if the delivery occurred there) and transported on ice to the field laboratory where they were stored in liquid nitrogen and later shipped to Cincinnati in dry shippers. Detailed laboratory methods are provided in the supplement to the primary results [7].

Enrolled women were visited weekly from the time of vaccination through 6 months postpartum to assess morbidity (self-reported fever, persistent cough, sore throat, nasal congestion, and myalgia) in the past week. If positive for fever and at least any one of the other symptoms in the past week, a mid-nasal swab was collected, stored in PrimeStore® Molecular Transport Medium (Longhorn Diagnostics LLC, Bethesda, MD) and later analyzed using real-time polymerase chain reaction (RT-PCR) for the presence of influenza. Maternal laboratory confirmed influenza was defined as maternal report of fever and at least one of the following: cough, sore throat, nasal congestion, or myalgia concurrent with a PCR-positive nasal swab. Infants were visited weekly from birth, and mothers were asked whether the infant had fever, cough, wheeze, difficulty breathing, or ear discharge on each day in the past week. If the mother reported the infant had any of these symptoms on any day, a mid-nasal swab was collected and later analyzed using RT-PCR for the presence of influenza. Episodes of infant influenza were defined as any one of fever, cough, wheeze, difficulty breathing, or ear discharge on at least 1 day concurrent with a PCR-positive nasal swab. Episodes were considered distinct if separated by at least 7 symptom-free days.

Other outcomes examined were influenza like illness in pregnancy and through 6 months postpartum in women, small-for-gestational-age (SGA), defined as having a birthweight less than the 10th percentile of the international Intergrowth-21 birthweight standard/reference at a specific gestational age [15], preterm defined as < 37 completed weeks gestation at birth, and mean difference between treatment groups in birthweight in grams and gestational age in weeks.

HAI titers were logarithmically transformed and geometric mean titers reported. Seroconversion was defined as a pre-vaccination HAI titer of 1:10 or less and a post-delivery maternal titer of 1:40 or larger, or a pre-vaccination titer greater than 1:10 and an increase in titer of 4 or more post-vaccination. Seroprotection was defined as HAI antibody titer ≥ 1:40.

Incidence rates for influenza were calculated by dividing the number of cases of illness by the number of days at risk by treatment group and compared using incidence risk ratios and 95% confidence intervals from mixed effects binomial regression models with log link to account for correlation between repeated morbidity measures. Days with symptoms and the subsequent 7 days were excluded from days at risk. The differences in mean birth weights and gestational ages between treatment groups were calculated using linear regression. Prevalence of LBW, preterm birth, and SGA by treatment group were compared using risk ratios and 95% confidence intervals from Poisson regression models with a log link and robust variance.

Verbal informed consent was obtained from all women who participated in the trial. The study was approved by the Institutional Review Boards (IRB) of the Nepal Health Research Council, the Institute of Medicine at Tribhuvan University, the Johns Hopkins Bloomberg School of Public Health (JHBSPH), and Cincinnati Children’s Hospital. IRBs at Seattle Children’s Hospital, the University of Washington, and George Washington University granted oversight to the IRB at JHBSPH. The trial was registered with ClinicalTrials.gov (NCT01034254). Funding for the study was provided by the Bill and Melinda Gates Foundation (grant 50274).

## RESULTS

A total of 3693 women were consented and vaccinated between April 2011 and September 2013, resulting in 3646 live births (2090 women, 2063 live births in cohort 1, 1603 women, 1583 live births in cohort 2). There was no difference between the placebo and vaccinated groups of women on a variety of demographic, socioeconomic, morbidity, and reproductive history characteristics at the time of enrollment, stratified by timing of vaccination in pregnancy (17–25 and 26–34 weeks) (Supplemental Table 1).

The incidence rate ratio (IRR) was not statistically significantly different from one for laboratory confirmed influenza between infants of women vaccinated earlier (IRR: 0.73 (95% confidence interval [CI]: 0.51, 1.05) or later in pregnancy (IRR: 0.63 (95% CI: 0.37, 1.08) in the cohorts combined, nor in each cohort (Table 1). The point estimates for the infant incidence rate ratios were more protective in the second cohort, which is consistent with greater protection in this cohort due to a better match between the vaccine and circulating virus, but there was no difference in protection by timing of vaccination in either cohort. The reduction in low birth weight in the combined cohorts was 17% among infants of mothers vaccinated between 17 and 25 weeks, although it was reduced by 10% in those vaccinated between 26 and 34 weeks, but this difference was not statistically significant (Table 1).

**Table 1.** Infant Influenza Incidence and Prevalence of Low Birth Weight by Cohort and Treatment Group

	Cohort 1		Cohort 2		Combined	
	17–25 Weeks	26–34 Weeks	17–25 Weeks	26–34 Weeks	17–25 Weeks	26–34 Weeks
**Infant Influenza**						
Vaccine cases (person-years)	44 (255.6)	16 (79.6)	8 (129.9)	6 (117.2)	52 (385.5)	22 (196.8)
Placebo cases (person-years)	50 (266.8)	19 (74.7)	21 (138.3)	15 (117.9)	71 (386.1)	34 (192.5)
Vaccine incidence^a^	172.2	201.0	61.6	51.2	134.9	111.8
Placebo incidence^a^	201.8	254.4	151.8	127.3	183.9	176.6
IRR (95% CI)	0.85 (0.56, 1.29)	0.79 (0.41, 1.53)	0.41 (0.18, 0.91)	0.40 (0.16, 1.02)	0.73 (0.51, 1.05)	0.63 (0.37, 1.08)
**Low Birthweight**						
Vaccine n/N (%)	142/606 (23.4)	51/201 (25.4)	63/296 (21.3)	59/277 (21.3)	205/902 (22.7)	110/478 (23.0)
Placebo n/N (%)	164/584 (28.1)	55/195 (28.2)	79/305 (25.9)	66/276 (23.9)	243/889 (27.3)	121/471 (25.7)
RR (95% CI)	0.83 (0.69, 1.01)	0.90 (0.65, 1.25)	0.82 (0.61, 1.10)	0.89 (0.65, 1.21)	0.83 (0.71, 0.98)	0.90 (0.72, 1.12)

Abbreviations: CI, confidence interval; IRR, incidence rate ratio; RR, relative risk.

^a^Incidence: cases/1000 person-years.

The geometric mean of the influenza HAI titers in cord blood was higher in infants of mothers vaccinated later than earlier in pregnancy for the first vaccine formulation used (245.0 (95% CI: 6.0, 962.2) versus 137.3 (95% CI: 83.2, 226.4) for A/H1N1/California, 43.1 (95% CI: 22.4, 82.8) versus 210.9 (95% CI: 77.5, 574.0) for A/H3N2/Perth and 94.3 (95% CI: 56.4, 157.7) versus 160.0 (95% CI: 93.9, 272.6) for B/Brisbane (V)), although these differences were not statistically significant (Table 2). With the second vaccine formulation used in the trial, geometric mean HAI titers were higher in infants whose mothers were vaccinated later in pregnancy, except for the A/H1N1/California antigen (198.3 (95% CI: 138.7, 283.4) versus 284.0 (95% CI: 195.5, 412.5)), but none of these differences were statistically significant. There were no statistically significant differences between early and later vaccination in pregnancy in the rise in geometric mean HAI titers from prevaccination to within 1 week postpartum in women, nor in the cord:maternal antibody transfer ratios (Table 2). Maternal seroconversion and seroprotection rates did not differ by timing of vaccination for the 2 vaccine formulae or specific antigens, but the sample sizes were small (Table 2). These comparisons included women in the placebo (N = 32) and vaccine (N = 20) groups who contracted influenza, which would have increased their antibody titers through natural infection.

**Table 2.** Geometric Mean Hemagglutination-Inhibition Antibodies (HAI) to Influenza Antigens in Cord Blood, Rise in Geometric Mean HAI Antibodies From Pre-vaccination to Within 1 Week Postpartum in Women, Infant-Mother Transfer Ratio, Maternal Seroprotection and Seroconversion by Vaccine Type and Timing of Vaccination

Vaccine 1	A/H1N1/California		A/H3N2/Perth		B/Brisbane (V)	
	Vaccine	Placebo	Vaccine	Placebo	Vaccine	Placebo
	Geometric Mean (95% CI)	Geometric Mean (95% CI)	Geometric Mean (95% CI)	Geometric Mean (95% CI)	Geometric Mean (95% CI)	Geometric Mean (95% CI)
**Cord blood**						
17–25 weeks	137.3 (83.2, 226.4)	19.8 (11.5, 33.9)	43.1 (22.4, 82.8)	14.0 (8.8, 22.2)	94.3 (56.4, 157.7)	24.6 (15.8, 38.4)
26–34 weeks	254.0 (67.0, 962.2)	18.3 (6.4, 52.3)	210.9 (77.5, 574.0)	14.1 (4.8, 41.4)	160.0 (93.9, 272.6)	18.3 (8.4, 40.2)
**Maternal increase**						
17–25 weeks	7.8 (4.3, 14.3)	0.9 (0.8, 1.2)	2.0 (1.4, 3.0)	1.0 (0.7, 1.4)	6.1 (4.2, 8.7)	1.7 (1.3, 2.3)
26–34 weeks	10.9 (4.1, 28.7)	1.3 (0.7, 2.4)	3.7 (1.0, 13.3)	1.4 (1.0, 1.9)	4.7 (1.7, 13.0)	1.8 (1.1, 3.0)
**Infant-mother ratio**						
17–25 weeks	1.0 (0.8, 1.4)	1.0 (0.8, 1.3)	0.9 (0.7, 1.0)	0.6 (0.5, 0.8)	0.9 (0.7, 1.1)	0.5 (0.4, 0.8)
26–34 weeks	1.0 (0.4, 2.3)	0.9 (0.6, 1.3)	0.7 (0.6, 0.9)	0.6 (0.5, 0.9)	1.1 (0.6, 1.9)	0.3 (0.2, 0.4)
**Seroprotection**	**n/N (%**)	**n/N (%**)	**n/N (%**)	**n/N (%**)	**n/N (%**)	**n/N (%**)
Pre-vaccine maternal						
17–25 weeks	5/20 (25.0)	9/24 (37.5)	8/20 (40.0)	8/24 (33.3)	4/20 (20.0)	8/24 (33.3)
26–34 weeks	3/9 (33.3)	3/8 (37.5)	6/9 (66.7)	3/8 (37.5)	4/9 (46.4)	4/8 (50.0)
**Post-vaccine maternal**						
17–25 weeks	18/20 (90.0)	7/24 (29.2)	13/20 (65.0)	8/24 (33.3)	20/20 (100.0)	16/24 (66.7)
26–34 weeks	8/9 (88.9)	4/8 (50.0)	8/9 (88.9)	3/8 (37.5)	9/9 (100.0)	7/8 (87.5)
**Maternal seroconversion**						
17–25 weeks	14/20 (70.0)	1/24 (4.2)	4/20 (20.0)	0/24 (0.0)	17/20 (85.0)	4/24 (16.7)
26–34 weeks	6/9 (66.7)	1/8 (12.5)	3/9 (33.3)	0/8 (0.0)	6/9 (66.7)	1/8 (12.5)
**Vaccine 2**	A/H1N1/California		A/H3N2/Victoria		B/Wisconsin (Y)	
	Vaccine	Placebo	Vaccine	Placebo	Vaccine	Placebo
	Geometric Mean (95% CI)	Geometric Mean (95% CI)	Geometric Mean (95% CI)	Geometric Mean (95% CI)	Geometric Mean (95% CI)	Geometric Mean (95% CI)
**Cord blood**						
17–25 weeks	284.0 (195.5, 412.5)	26.2 (13.3, 51.6)	284.6 (183.7, 440.9)	55.3 (30.4, 100.5)	274.5 (198.2, 380.2)	80.0 (53.5, 119.6)
26–34 weeks	198.3 (138.7, 283.4)	27.9 (18.7, 41.6)	319.3 (229.9, 443,5)	38.4 (26.0, 56.7)	372.6 (277.9, 499.5)	66.9 (51.7, 86.6)
**Maternal increase**						
17–25 weeks	5.4 (3.3, 8.9)	0.8 (0.6, 1.1)	6.9 (4.7, 10.3)	1.2 (0.6, 2.1)	2.5 (2.1, 3.1)	1.0 (0.7, 1.3)
26–34 weeks	6.3 (4.4, 9.0)	1.0 (0.9, 1.1)	10.4 (7.4, 14.8)	1.4 (1.0, 2.0)	2.5 (1.9, 3.3)	1.2 (1.0, 1.4)
**Infant-mother ratio**						
17–25 weeks	1.6 (1.1, 2.4)	1.2 (0.8, 1.8)	1.2 (0.9, 1.5)	0.9 (0.6, 1.6)	0.8 (0.6, 1.0)	0.5 (0.3, 0.8)
26–34 weeks	1.2 (0.9, 1.5)	1.1 (1.0, 1.3)	1.3 (1.1, 1.5)	0.8 (0.6, 1.0)	0.8 (0.6, 1.0)	0.4 (0.3, 0.5)
**Seroprotection**	n/N (%)	n/N (%)	n/N (%)	n/N (%)	n/N (%)	n/N (%)
**Pre-vaccine maternal**						
17–25 weeks	15/29 (51.7)	11/27 (40.7)	16/29 (55.2)	16/27 (59.3)	28/29 (96.6)	27/27 (100.0)
26–34 weeks	22/49 (44.9)	20/42 (47.6)	23/49 (46.9)	23/42 (54.8)	48/49 (98.0)	41/42 (97.6)
**Post-vaccine maternal**						
17–25 weeks	27/29 (93.1)	8/27 (29.6)	29/29 (100.0)	18/27 (66.7)	29/29 (100.0)	27/27 (100.0)
26–34 weeks	46/49 (93.9)	20/42 (47.6)	48/49 (98.0)	27/42 (64.3)	49/49 (100.0)	42/42 (100.0)
**Maternal seroconversion**						
17–25 weeks	16/29 (55.2)	0/27 (0.0)	22/29 (75.9)	4/27 (14.8)	11/29 (37.9)	2/27 (7.4)
26–34 weeks	35/49 (71.4)	1/42 (2.4)	40/49 (81.6)	6/42 (14.3)	22/49 (44.9)	2/42 (4.8)

Abbreviation: CI, confidence interval.

Corresponding to the point estimates of the reduction in LBW being greater in infants of women who were vaccinated earlier in pregnancy, the mean difference in birth weight between the infants of vaccinated and placebo mothers was greater for those vaccinated earlier in pregnancy (Table 3). However, none of these differences between early and late vaccination were statistically significant. Similarly, there were no statistically significant differences in a variety of other secondary outcomes (ILI in women and infants, maternal laboratory confirmed influenza, SGA, preterm, stillbirths, infant mortality, and congenital defects) (Table 3).

**Table 3.** Incidence Rate Ratios, Relative Risks, and Mean Differences Between Vaccine and Placebo for Other Outcomes of the Maternal Influenza Vaccination by Gestational Age at Vaccination

	Cohort 1		Cohort 2		Combined	
	17–25 Weeks	26–34 Weeks	17–25 Weeks	26–34 Weeks	17–25 Weeks	26–34 Weeks
**IRR (95% CI**)	**IRR (95% CI**)	**IRR (95% CI**)	**IRR (95% CI**)	**IRR (95% CI**)	**IRR (95% CI**)
ILI pregnancy	0.85 (0.60, 1.21)	0.58 (0.22, 1.53)	0.57 (0.33, 1.00)	0.65 (0.30, 1.42)	0.76 (0.56, 1.02)	0.62 (0.33, 1.15)
ILI postpartum	0.96 (0.64, 1.43)	1.13 (0.64, 2.02)	0.82 (0.44, 1.55)	0.53 (0.25, 1.14)	0.92 (0.66, 1.30)	0.86 (0.54, 1.37)
All ILI	0.89 (0.67, 1.18)	0.95 (0.58, 1.57)	0.69 (0.44, 1.08)	0.58 (0.33, 1.03)	0.83 (0.65, 1.05)	0.77 (0.52, 1.12)
Flu pregnancy	0.37 (0.13, 1.03)	0.94 (0.13, 6.68)	1.31 (0.41, 4.18)	1.57 (0.38, 6.52)	0.62 (0.30, 1.31)	1.32 (0.42, 4.14)
Flu postpartum	0.52 (0.20, 1.35)	1.46 (0.25, 8.71)	NA	0.20 (0.02, 1.67)	0.62 (0.25, 1.54)	0.56 (0.16, 1.90)
All flu	0.44 (0.22, 0.88)	1.21 (0.33, 4.47)	1.50 (0.49, 4.58)	0.73 (025, 2.07)	0.62 (0.35, 1.10)	0.89 (0.39, 2.00)
ILI infants	1.04 (0.86, 1.27)	1.01 (0.75, 1.37)	0.73 (0.55, 0.96)	1.04 (0.79, 1.36)	0.92 (0.78, 1.08)	1.03 (0.84, 1.26)
	**RR (95% CI**)	**RR (95% CI**)	**RR (95% CI**)	**RR (95% CI**)	**RR (95% CI**)	**RR (95% CI**)
Preterm	0.97 (0.77, 1.24)	0.84 (0.51, 1.38)	0.94 (0.67, 1.34)	0.79 (0.52, 1.20)	0.97 (0.79, 1.18)	0.81 (0.59, 1.11)
SGA	0.86 (0.73, 1.00)	0.97 (0.77, 1.24)	0.95 (0.75, 1.20)	1.05 (0.83, 1.32)	0.88 (0.78, 1.01)	1.02 (0.86, 1.20)
Stillbirths	1.35 (0.62, 2.92)	0.72 (0.23, 2.23)	0.92 (0.34, 2.51)	1.16 (0.36, 3.78)	1.18 (0.63, 2.21)	0.90 (0.40, 2.03)
Infant deaths	0.93 (0.47, 1.83)	1.00 (0.36, 2.82)	2.22 (1.02, 4.84)	0.96 (0.51, 1.82)	1.36 (0.82, 2.24)	0.98 (0.57, 1.69)
Congenital defects	0.86 (0.31, 2.36)	2.94 (0.60, 14.42)	0.86 (0.23, 3.17)	0.97 (0.20, 4.77)	0.86 (0.39, 1.90)	1.75 (0.59, 5.20)
	**Mean (95% CI**)	**Mean (95% CI**)	**Mean (95% CI**)	**Mean (95% CI**)	**Mean (95% CI**)	**Mean (95% CI**)
Birthweight difference (g)	34.2 (−17.5, 85.8)	10.9 (−77.5, 99.2)	77.1 (5.9, 148.2)	45.6 (−30.5, 121.7)	47.7 (5.9, 89.6)	30.5 (−27.4, 88.4)
Gestational age difference (wk)	−0.07 (−0.34, 0.21)	0.15 (−0.26, 0.55)	0.04 (−0.29, 0.37)	0.29 (−0.05, 0.63)	−0.03 (−0.25, 0.18)	0.23 (−0.03, 0.49)

Abbreviations: CI, confidence interval; g, grams; ILI, influenza-like illness; IRR, incidence rate ratios; NA, not applicable; RR, relative risks; SGA, small-for-gestational-age; wk, week.

## DISCUSSION

The primary outcomes of the original trial, maternal ILI, infant laboratory-confirmed influenza, and LBW were significantly reduced by maternal vaccination across the 2 cohorts combined. In this subanalysis of the maternal influenza vaccination trial, we did not find any statistical evidence that the impact of the vaccine on maternal and infant antibody responses or health outcomes differed by the timing of vaccination during pregnancy. There was some suggestion from point estimates that antibody levels in cord blood were higher for most antigens in the vaccine among infants whose mothers were vaccinated later in pregnancy, but the numbers of samples tested for antibodies was small, limiting the power to detect meaningful differences. Additionally, there was a somewhat higher birth weight among infants whose mothers were vaccinated earlier in pregnancy, but none of these differences between early and late vaccination were statistically significant.

Many studies have compared first to second and third trimester safety outcomes for influenza vaccination, but few studies have compared efficacy outcomes between second and third trimester influenza vaccination. An observational study in Australia found a 33% reduction in hospitalizations for respiratory illness among newborns of women vaccinated in the third trimester of pregnancy but no effect if vaccinated earlier in pregnancy [16]. An observational study in Nicaragua found reductions in preterm birth if vaccinated in the second and third trimesters of 13% and 34%, respectively, and a 20% and 36% reduction in LBW in infants of women vaccinated in the second and third trimester, respectively [17]. A retrospective analysis of 7 Vaccine Safety Datalink sites in the United States did not find any association between vaccine exposure and preterm and SGA overall, nor by trimester [18]. Sperling et al. examined the immunogenicity of influenza vaccine in pregnancy and found seroconversion rates to be similar in the second trimester (13 to <28 weeks) compared to early third trimester (28 to <34 weeks) [19]. We were unable to identify any randomized trials that have examined efficacy of infant health outcomes by timing of influenza vaccination, although we identified 2 randomized trials that examined antibody response to pandemic H1N1 vaccination in cord blood by gestational age at vaccination [10, 11]. One found lower antibody response at birth for infants of mothers vaccinated earlier in pregnancy [10], but the other trial did not [11]. Recent prospective observational studies of tetanus-diphtheria-acellular pertussis (Tdap) immunization in pregnancy have evaluated the effect of second versus third trimester vaccination on cord blood antibodies and infant seropositivity rates [20, 21]. These studies found that early second trimester Tdap immunization increases neonatal antibodies, suggesting that for Tdap, a broader immunization window would maximize infant protection from pertussis in both full-term and preterm infants.

Physiogically, transplacental antibody transfer occurs starting in the second trimester but increases significantly in the third trimester. Therefore, it would be expected that antibody titers may be higher in the cord blood of infants whose mothers were vaccinated later in pregnancy. However, these infants have had the benefit of higher maternal antibody levels and maternal protection from illness for less time during pregnancy, perhaps explaining the slightly higher impact of second trimester influenza vaccination on mean birth weights and greater reduction in LBW relative to the vaccine’s impact in the third trimester.

Strengths of this study include a large individually randomized population-based controlled trial design with vaccinations occurring over 2 annual cohorts. In addition, the design included randomization stratified on timing of vaccination during pregnancy, allowing this analysis to retain the advantages of the randomized design within these strata. The availability of antibody measurements to the vaccine antigens was also helpful in further explaining the health outcome findings.

Limitations of the study include self-report of morbidity, although influenza in mothers and infants was confirmed through analysis of nasal swabs by RT-PCR. However, maternal influenza was only confirmed for febrile illness, whereas the infant influenza was confirmed on the basis of any ILI symptom. Although there was some indication from point estimates of differences in the impact of timing of vaccination on cord blood antibodies and birth weight, these difference were not statistically significant. A larger sample size might have been able to strengthen the evidence for these differences, especially with respect to outcomes HAI antibody titers. However, the actual differences are not of that much public health importance. Another limitation is the lack of gold standard gestational age measurements using ultrasound. However, the 5-weekly surveillance for pregnancy and confirmation by pregnancy test reduced the recall period of date of LMP. This approach to gestational age estimation was recently validated and found quite accurate against early ultrasound measurements in a similar population in Bangladesh [22].

The public health importance of these findings is that vaccination in either the second or third trimester of pregnancy is equally likely to provide protection from influenza. In many low income settings where women often present for antenatal care infrequently or late influenza immunization administered anytime in the 2nd or 3rd trimester of pregnancy could provide protection. This makes the logistics of implementing a maternal influenza immunization strategy more feasible in resource-limited settings.

## Supplementary Data

Supplementary materials are available at *Clinical Infectious Diseases* online. Consisting of data provided by the authors to benefit the reader, the posted materials are not copyedited and are the sole responsibility of the authors, so questions or comments should be addressed to the corresponding author.

Supplementary Table

## Notes

***Authors’ Contributions***. J. K., M. C. S., J. M. T., J. A. E., and J. K. contributed to the design of the study. M. C. S. conceived of the study and secured funding for the project. S. K. K., S. C. L., L. S., J. M. T., L. C. M., and J. K. supervised the conduct of the study in the field. J. K. drafted the manuscript, served as the study statistician, and J. K. and L. C. M. designed/implemented the data analysis. H. Y. C. assisted in study implementation and cord blood collection. N. K. provided data analysis support. All authors read and approved the final manuscript.

***Disclaimer.*** The funding agency had no role in the design, conduct, analysis, and interpretation of the findings, or in the decision to publish.

***Financial support.*** This work was supported by a grant from the Bill and Melinda Gates Foundation (grant OPP50274).

***Potential conflicts of interest.*** H. Y. C. has received research support from GSK, Sanofi Pasteur, and Novavax. J. E. has been a consultant for Pfizer, a member of a Data Safety Monitoring Board for GlaxoSmithKline (GSK) influenza antiviral studies, and her institution has received research support for clinical studies from GSK, Gilead, Chimerix, and Roche. All authors have submitted the ICMJE Form for Disclosure of Potential Conflicts of Interest. Conflicts that the editors consider relevant to the content of the manuscript have been disclosed.
