# Supplementary material for: Impact of Timing of Influenza Vaccination in Pregnancy on Transplacental Antibody Transfer, Influenza Incidence, and Birth Outcomes: A Randomized Trial in Rural Nepal
Source: Clin Infect Dis. 2018 Feb 14;67(3):334–40. doi: 10.1093/cid/ciy090 (PMC6051462; doi:10.1093/cid/ciy090)
Supplement: Supplementary Table [file ciy090_suppl_supplementary_table.docx]

**Supplemental Table 1. Baseline maternal characteristics at time of vaccination by cohort and gestational age at vaccination**

| **Cohort 1** | | **Cohort 2** | | **Combined** | |  |
| --- | --- | --- | --- | --- | --- | --- |
| 17-25 weeks gestation | **Placebo (N=768)** | **Vaccine (N=774)** | **Placebo (N=385)** | **Vaccine (N=379)** | **Placebo (N=1153)** | **Vaccine (N=1153)** |
| 26-34 weeks gestation | **Placebo (N=273)** | **Vaccine (N=275)** | **Placebo (N=420)** | **Vaccine (N=419)** | **Placebo (N=693)** | **Vaccine (N=694)** |
|  | **Mean (SD)** | **Mean (SD)** | **Mean (SD)** | **Mean (SD)** | **Mean (SD)** | **Mean (SD)** |
| **Maternal Age (years)** |  |  |  |  |  |  |
| 17-25 weeks gestation | 23.0 (4.6) | 23.2 (4.4) | 23.1 (4.6) | 23.4 (5.0) | 23.0 (4.6) | 23.3 (4.7) |
| 26-34 weeks gestation | 23.1 (4.7) | 23.2 (4.7) | 23.4 (4.7) | 23.3 (4.8) | 23.3 (4.7) | 23.2 (4.8) |
| **Gestational Age at vaccination (weeks)** |  |  |  |  |  |  |
| 17-25 weeks gestation | 19.4 (2.6) | 19.2 (2.6) | 21.6 (2.6) | 21.6 (2.6) | 20.2 (2.8) | 20.0 (2.8) |
| 26-34 weeks gestation | 29.8 (2.6) | 29.8 (2.5) | 28.6 (2.1) | 28.7 (2.1) | 29.1 (2.4) | 29.2 (2.3) |
| **Gestational Age at birth (weeks)** |  |  |  |  |  |  |
| 17-25 weeks gestation | 39.2 (3.9) | 39.2 (2.9) | 39.2 (2.4) | 39.2 (2.7) | 39.3 (2.8) | 39.2 (2.8) |
| 26-34 weeks gestation | 39.8 (4.1) | 39.7 (2.7) | 39.4 (2.8) | 39.6 (2.5) | 39.5 (3.4) | 39.7 (2.6) |
| **Weeks between vaccination and birth** |  |  |  |  |  |  |
| 17-25 weeks gestation | 19.8 (3.9) | 20.0 (4.1) | 17.5 (3.7) | 17.7 (3.7) | 19.0 (4.0) | 19.2 (4.1) |
| 26-34 weeks gestation | 9.8 (3.8) | 10.0 (3.5) | 10.9 (3.5) | 10.9 (3.6) | 10.5 (3.6) | 10.5 (3.5) |
| **Parity** |  |  |  |  |  |  |
| 17-25 weeks gestation | 1.2 (1.6) | 1.2 (1.5) | 1.0 (1.4) | 1.1 (1.6) | 1.1 (1.5) | 1.1 (1.6) |
| 26-34 weeks gestation | 1.1 (1.5) | 1.1 (1.5) | 1.0 (1.3) | 1.0 (1.5) | 1.1 (1.4) | 1.0 (1.5) |
| **Diastolic Blood Pressure (mmHg)** |  |  |  |  |  |  |
| 17-25 weeks gestation | 99.7 (9.9) | 100.4 (9.9) | 100.5 (10.2) | 100.2 (9.3) | 99.9 (10.0) | 100.3 (9.7) |
| 26-34 weeks gestation | 100.8 (9.2) | 100.4 (9.7) | 101.0 (9.4) | 101.2 (9.6) | 100.9 (9.3) | 100.9 (9.6) |
| **Systolic Blood Pressure (mmHg)** |  |  |  |  |  |  |
| 17-25 weeks gestation | 66.1 (8.5) | 66.8 (9.4) | 6.9 (9.2) | 66.6 (8.2) | 66.4 (8.8) | 66.7 (9.0) |
| 26-34 weeks gestation | 67.0 (8.9) | 66.4 (9.6) | 67.2 (8.4) | 67.3 (9.0) | 67.1 (8.6) | 66.9 (9.2) |
| **Height (cm)** |  |  |  |  |  |  |
| 17-25 weeks gestation | 151.5 (5.7) | 151.5 (5.5) | 151.8 (5.6) | 151.7 (5.1) | 151.6 (5.6) | 151.6 (5.4) |
| 26-34 weeks gestation | 151.8 (5.6) | 152.1 (5.8) | 151.7 (5.5) | 151.4 (5.5) | 151.7 (5.5) | 151.7 (5.6) |
| **Weight (kg)** |  |  |  |  |  |  |
| 17-25 weeks gestation | 46.9 (7.2) | 47.8 (7.8) | 47.5 (6.9) | 47.6 (7.0) | 47.1 (7.1) | 47.7 (7.5) |
| 26-34 weeks gestation | 51.1 (6.3) | 51.9 (7.7) | 49.1 (7.2) | 49.3 (7.9) | 49.9 (6.9) | 50.3 (7.9) |
| **BMI ( kg/m^2^)** |  |  |  |  |  |  |
| 17-25 weeks gestation | 20.4 (2.8) | 20.8 (2.9) | 20.6 (2.7) | 20.7 (2.8) | 20.5 (2.7) | 20.7 (2.9) |
| 26-34 weeks gestation | 22.1 (2.4) | 22.4 (2.9) | 21.3 (2.7) | 21.4 (3.0) | 21.7 (2.6) | 21.8 (3.0) |
|  |  |  |  |  |  |  |
|  | **n (%)** | **n (%)** | **n (%)** | **n (%)** | **n (%)** | **n (%)** |
| **BMI categories 17-25 weeks gestation** |  |  |  |  |  |  |
| <16 | 14 (1.8) | 14 (1.8) | 6 (1.4) | 5 (1.2) | 20 (1.7) | 19 (1.6) |
| 16-<18·5 | 163 (21.1) | 145 (18.5) | 81 (19.2) | 79 (19.7) | 244 (20.4) | 224 (18.9) |
| 18·5-<25 | 547 (70.8) | 571 (72.9) | 309 (73.1) | 294 (73.1) | 856 (71.6) | 865 (73.0) |
| 25+ | 49 (6.3) | 53 (6.8) | 27 (6.4) | 24 (6.0) | 76 (6.4) | 77 (6.5) |
| **BMI categories 26-34 weeks gestation** |  |  |  |  |  |  |
| <16 | 0 | 0 | 3 (0.8) | 3 (0.8) | 3 (0.5) | 3 (0.5) |
| 16-<18·5 | 13 (4.9) | 14 (5.4) | 58 (15.2) | 49 (12.4) | 71 (11.0) | 63 (9.6) |
| 18·5-<25 | 218 (82.9) | 202 (77.4) | 288 (75.4) | 295 (74.9) | 506 (78.5) | 497 (75.9) |
| 25+ | 32 (12.2) | 45 (17.2) | 33 (8.6) | 47 (11.9) | 65 (10.1) | 92 (14.1) |
| **No maternal education** |  |  |  |  |  |  |
| 17-25 weeks gestation | 297 (41.1) | 331 (45.0) | 126 (35.7) | 134 (38.3) | 423 (39.3) | 465 (42.9) |
| 26-34 weeks gestation | 130 (49.2) | 115 (43.1) | 174 (43.4) | 164 (42.5) | 304 (45.7) | 279 (42.7) |
| **Nulliparous** |  |  |  |  |  |  |
| 17-25 weeks gestation | 304 (39.6) | 301 (38.9) | 190 (49.4) | 161 (42.6) | 494 (42.8) | 462 (40.1) |
| 26-34 weeks gestation | 113 (41.9) | 112 (41.0) | 182 (43.3) | 185 (44.2) | 295 (42.8) | 297 (42.9) |
| **History of child deaths*** |  |  |  |  |  |  |
| 17-25 weeks gestation | 79 (18.0) | 63 (14.2) | 22 (10.6) | 28 (12.4) | 101 (15.6) | 91 (13.6) |
| 26-34 weeks gestation | 23 (15.7) | 23 (15.5) | 29 (14.0) | 16 (7.8) | 52 (14.7) | 39 (11.0) |
| **History of stillbirths*** |  |  |  |  |  |  |
| 17-25 weeks gestation | 37 (8.0) | 29 (6.1) | 5 (2.6) | 6 (2.8) | 42 (6.4) | 35 (5.1) |
| 26-34 weeks gestation | 8 (5.1) | 6 (3.7) | 9 (3.8) | 14 (6.0) | 17 (4.3) | 20 (5.1) |

* Among women with at least one prior live birth

17-25 weeks gestation:

Includes 1 pregnancy in cohort 1, 0 placebo and 1 in vaccine group, who were vaccinated within 14 days of delivery

Includes 1 pregnancy in cohort 2, 0 placebo and 1 in vaccine group, who were vaccinated within 14 days of delivery

26-34 weeks gestation:

Includes 8 pregnancies in cohort 1, 4 placebo and 4 in vaccine group, who were vaccinated within 14 days of delivery.

Includes 9 pregnancies in cohort 2, 6 placebo and 3 in vaccine group (vaccine group: 2 infants, one twin pair), who were vaccinated within 14 days of delivery
